# Novel Targets for Old and Diseased Hearts

**DOI:** 10.3390/ijms23126627

**Published:** 2022-06-14

**Authors:** Erica Rurali, Giulio Pompilio, Serena Zacchigna

**Affiliations:** 1 Unità di Biologia Molecolare e Medicina Rigenerativa, Centro Cardiologico Monzino IRCCS, 20138 Milan, Italy; giulio.pompilio@cardiologicomonzino.it; 2 Dipartimento di Scienze Biomediche Chirurgiche Odontoiatriche, Università degli Studi di Milano, 20122 Milan, Italy; 3 International Centre for Genetic Engineering and Biotechnology (ICGEB), 34149 Trieste, Italy; 4 Department of Medicine, Surgery and Health Sciences, University of Trieste, 34149 Trieste, Italy

In this Special Issue we cover a selection of original articles and reviews devoted to the definition of novel molecular targets in cardiovascular diseases, which not only deepen our knowledge on the pathogenesis of the diseases under study, but potentially pave the way to novel diagnostic tools and therapeutic approaches.

In the complicated and not yet fully elucidated pathophysiology of heart failure (HF), oxidative stress is a major player. The manuscript by Tomin et al. proposes novel oxidative stress-related molecular targets, using an integrated mass spectrometry-based approach to study differential protein expression and oxidation [1]. They then confirmed their results by using an in vitro model of human cardiomyocytes cultured in a controlled oxygen atmosphere. Through this integrated analysis, the Authors identified a large dataset of dysregulated elements in the left ventricle of failing human hearts vs. controls, which certainly represent a useful reservoir for finding molecules involved in HF pathogenesis [1]. In addition to a lower level of the glutathione-to-glutathione disulfide ratio (GSH/GSSG), the Authors identified a number of proteins involved in myocyte contractile machinery and some glycolytic enzymes that are more oxidized in failing hearts. Oxygen concentration also affects extracellular matrix remodeling, glycolysis, and ion transport, due to changes in the abundance of some target proteins [1]. Therefore, in addition to providing relevant information on HF pathogenesis, this manuscript offers a rich dataset for further prognostic and mechanistic studies of proteome remodeling in this pathological context.

Moving to another complex disease such as diabetes, the study by D’Alessandra et al. showed for the first time that the transcriptional signature of bone marrow-derived CD34+ hematopoietic stem cells (HSPC) induced by diabetes is predictive of the dysfunction of their progeny [2]. Specifically, by sequencing CD34+ HSPC total RNA obtained from diabetic and non-diabetic patients undergoing coronary artery bypass surgery, the Authors identified 139 differentially expressed genes that represent novel targets for further investigation in this pathological context. Indeed, CD34+ HSPC obtained from diabetic patients with coronary artery disease showed altered expression of several genes encoding for chemokines and inflammatory cytokines, which predict the behavior of CD34+ progeny [2]. Considering the strong relationship between the progeny of HSPC and atherosclerotic plaque formation, these findings could pave the way for the discovery of new targets responsible for the onset of diabetes-related micro- and macrovascular complications that could eventually result in organ failure.

Finally, the review by Junco-Vincente and co-workers provides an update on the latest developments on the genetic and molecular determinants involved in the pathogenesis of bicuspid aortic valve (BAV) and its associated aortopathy [3]. At present, there are no validated biomarkers to be used in the management of patients affected by bicuspid aortopathy, while they would be extremely relevant to decide about the optimal timing for surgical replacement, monitor disease progression and assess patient’s risk profile and prognosis. They would also allow anticipating the diagnosis. While the disease is currently diagnosed at a later stage, when the aortic structure is already compromised, early diagnosis could allow implementing targeted therapies and prevent disease progression. The review offers some perspective on some molecular targets that could be exploited therapeutically to prevent disease progression, including Neurogenic locus notch homolog protein 1 (NOTCH1) and Protein kinase B (AKT) signaling pathways [3]. Yet, to what extent these interventions could lead to a real improvement in the management of these patients remains to be established.

Turning to the implementation of a diagnostic tool, the review by Stadiotti et al. [4] succeeds in creating a useful parallel between endurance athletes and Arrhythmogenic Cardiomyopathy (ACM) patients opening the way to the exploitation of a diagnostic tool to a novel population. The innovative point of view of the Authors leads the review starting from an in-depth description of the “Extreme Exercise Hypothesis” that correlates exercise training volumes and health risk in healthy subjects highlighting that some athletes could develop sudden cardiac death and myocardial infarction, basically due to changes in myocardial structure and function, and continuing with the description of the modulation of pathological biomarkers induced by exercise [4]. The clinical cardiac phenotype of patients with ACM is compared with the exercise-induced cardiac remodeling observed in some endurance athletes and several commonalities, such as left ventricular size modifications, myocardial inflammation, cardiac oxidative stress, and electrical abnormality development, emerged. Finally, considering the presence of circulating autoantibodies against Desmoglein-2 in ACM patients, the Authors raise the question about the possible presence of these antibodies even in endurance athletes who develop cardiomyopathy in respect to those that develop only transient myocardial modifications [4]. In case this assumption is well-founded, a simple blood test would allow clinicians to identify athletes at risk of serious cardiac damage among all those who practice endurance sports. Immunomodulatory drugs might be of help for athletes at risk, although additional studies need to be performed to validate this hypothesis.

More concrete evidence of novel targets to be exploited therapeutically for vascular disorders are provided by two additional works that focus on two major pathogenetic mechanisms of vessel disease, namely thrombosis and arterial restenosis [5].

Thrombosis, the complete occlusion of vessels, is often consequent to uncontrolled platelet activation, and leads to life-threatening ischemia in many organs. Available drugs able to interfere with platelet aggregation are associated with increased risk of hemorrhage, warranting the development of effective, yet safer, anti-platelet drugs. The work by Barrachina et al. has identified a compound, called Idelalisib, which potently inhibits platelet adhesion and aggregation by blocking Phosphoinositide 3-kinases (PI3K), but shows minimal bleeding effects in mice, compared to standard anti-platelet therapy, such as aspirin and clopidogrel, in mouse models [5,6].

Recurrence of arterial stenosis (restenosis) after percutaneous transluminal coronary angioplasty (PTCA) is a frequent maladaptive response, occurring in up to 50% of patients, often requiring target vessel revascularization at one year, and associated with increased morbidity, mortality and health care costs. Pan and colleagues add a piece of evidence on the pathogenesis of the disease and provide novel insight into the role of phosphoglycerate kinase 1 (PGK1) in the regulation of vascular smooth muscle cell proliferation and neointima formation in hypoxic conditions [6]. Using both primary cell cultures and a rat carotid artery injury model, they showed that PGK1 is rapidly induced early after balloon angioplasty and that its inhibition effectively reduced neointima formation, thus providing evidence that a short-term intervention targeting early response genes could be as effective in preventing restenosis as long-term drug release [6].

Cardiovascular diseases represent the first cause of morbidity and mortality worldwide. Yet, the drugs that are used to treat these diseases are often quite old, with only few advances introduced in the cardiovascular pharmacopeia over the last 30 years. The same holds true for biomarkers able to predict disease progression and patient prognosis. Investing in basic science and understanding the molecular mechanisms responsible for the onset and progression of cardiovascular diseases appear essential if we are to have the tools in hand to combat the number one killer of humanity.
